# Outbreaks Associated with Treated Recreational Water — United States, 2015–2019

**DOI:** 10.15585/mmwr.mm7020a1

**Published:** 2021-05-21

**Authors:** Michele C. Hlavsa, Samaria K. Aluko, Allison D. Miller, John Person, Megan E. Gerdes, Sooji Lee, Joseph P. Laco, Elizabeth J. Hannapel, Vincent R. Hill

**Affiliations:** ^1^Division of Foodborne, Waterborne, and Environmental Diseases, National Center for Emerging and Zoonotic Infectious Diseases, CDC; ^2^Oak Ridge Institute for Science and Education, Oak Ridge, Tennessee; ^3^Division of Bacterial Diseases, National Center for Immunization and Respiratory Diseases; ^4^Division of Environmental Health Science and Practice, National Center for Environmental Health, CDC.

Outbreaks associated with treated recreational water can be caused by pathogens or chemicals in aquatic venues such as pools, hot tubs, water playgrounds, or other artificially constructed structures that are intended for recreational or therapeutic purposes. For the period 2015–2019, public health officials from 36 states and the District of Columbia (DC) voluntarily reported 208 outbreaks associated with treated recreational water. Almost all (199; 96%) of the outbreaks were associated with public (nonbackyard) pools, hot tubs, or water playgrounds. These outbreaks resulted in at least 3,646 cases of illness, 286 hospitalizations, and 13 deaths. Among the 155 (75%) outbreaks with a confirmed infectious etiology, 76 (49%) were caused by *Cryptosporidium* (which causes cryptosporidiosis, a gastrointestinal illness) and 65 (42%) by *Legionella* (which causes Legionnaires’ disease, a severe pneumonia, and Pontiac fever, a milder illness with flu-like symptoms). *Cryptosporidium* accounted for 2,492 (84%) of 2,953 cases resulting from the 155 outbreaks with a confirmed etiology. All 13 deaths occurred in persons affected by a Legionnaires’ disease outbreak. Among the 208 outbreaks, 71 (34%) were associated with a hotel (i.e., hotel, motel, lodge, or inn) or a resort, and 107 (51%) started during June–August. Implementing recommendations in CDC’s Model Aquatic Health Code (MAHC) (*1*) can help prevent outbreaks associated with treated recreational water in public aquatic venues.

An outbreak associated with recreational water is the occurrence of similar illness in two or more persons whose illnesses are epidemiologically linked by location and time of exposure to 1) recreational water or 2) pathogens or chemicals aerosolized or volatilized into the air from recreational water. Public health officials in U.S. jurisdictions (the 50 states, DC, U.S. territories, and freely associated states) voluntarily report outbreaks to CDC via the National Outbreak Reporting System. This report examines data on outbreaks that were associated with treated recreational water and reported by February 4, 2021, and for which the first illness occurred during 2015–2019. Data on each outbreak include earliest illness onset date, count of cases of illness, counts of hospitalizations and deaths, etiology, and setting (e.g., hotel) and venue (e.g., pool, hot tub, or water playground) of the outbreak exposure. This activity was reviewed by CDC and was conducted consistent with applicable federal law and CDC policy.*

For the period 2015–2019, public health officials from 36 states^†^ and DC reported 208 outbreaks associated with treated recreational water, which resulted in at least 3,646 cases of illness (Table), 286 hospitalizations, and 13 deaths. Almost all (199; 96%) of the outbreaks were associated with public pools, hot tubs, or water playgrounds. Etiology was confirmed for 155 (75%) of the 208 outbreaks. These 155 outbreaks were all caused by pathogens and resulted in at least 2,953 (81%) cases and 266 (93%) hospitalizations. The 76 (49%) outbreaks caused by *Cryptosporidium* accounted for 2,492 (84%) of the 2,953 cases and 82 (31%) of the 266 hospitalizations. Unlike other pathogens, which caused outbreaks resulting in <100 cases of illness, *Cryptosporidium* caused outbreaks resulting in >100 cases of illness. The four such cryptosporidiosis outbreaks resulted in a total of 1,380 cases; the largest outbreak resulted in 638 cases. The 65 (42%) outbreaks caused by *Legionella* accounted for 354 (12%) of the 2,953 cases and 177 (67%) of the 266 hospitalizations. Four outbreaks caused by *Legionella* accounted for 178 (6%) of the 2,953 cases and 54 (20%) of the 266 hospitalizations. All 13 deaths occurred in persons affected by a Legionnaires’ disease outbreak. Among the 53 outbreaks with a nonconfirmed (i.e., suspected or unknown) etiology, 20 (38%) were suspected to be caused by chemical etiologies (e.g., excess chlorine, one or more disinfection byproducts, or altered pool chemistry) (Table).

**TABLE:** Outbreaks associated with treated recreational water,* by etiology — National Outbreak Reporting System, United States, 2015–2019

Etiology	No. of outbreaks (%)^†^	No. of cases (%)^†^	Median no. of cases (minimum–maximum)
**Total**	**208 (100)**	**3,646 (100)**	**5 (2–638)**
**Confirmed infectious etiology**	**155 (75)**	**2,953 (81)**	**4 (2–638)**
**Bacterium**	**72 (35)**	**386 (11)**	**2 (2–92)**
*Legionella*	65 (31)	354 (10)	2 (2**–**92)
Shiga toxin–producing *Escherichia coli*	4 (2)	17 (<1)	4.5 (2**–**6)
*Campylobacter*	1 (<1)	4 (<1)	—**^§^**
Nontuberculous mycobacteria	1 (<1)	9 (<1)	—
*Shigella*	1 (<1)	2 (<1)	—
**Parasite**	**80 (38)**	**2,503 (69)**	**8.5 (2–638)**
*Cryptosporidium*	76 (37)	2,492 (68)	9.5 (2**–**638)
*Giardia*	3 (1)	9 (<1)	3 (2**–**4)
*Acanthamoeba*	1 (<1)	2 (<1)	—
**Virus**	**3 (1)**	**64 (2)**	**14 (14–36)**
Norovirus	3 (1)	64 (2)	14 (14**–**36)
**Nonconfirmed^¶^**	**53 (25)**	**693 (19)**	**8 (2–94)**

* Treated recreational water is water in a pool, hot tub, water playground, or other artificially constructed structure that is intended for recreational or therapeutic purposes. Outbreaks are the occurrence of similar illness in two or more persons who are epidemiologically linked by location and time of exposure to 1) treated recreational water or 2) pathogens or chemicals that were aerosolized or volatilized into the air from treated recreational water.

^†^ Percentages do not sum to 100 because of rounding.

^§^ Dashes indicate median not provided because only one outbreak was reported for that specific etiology.

^¶^ Includes outbreaks with the following reported etiologies: suspected chemical (e.g., excess chlorine, one or more disinfection byproducts, or altered pool chemistry) for 20 outbreaks (10%), unknown for 12 (6%), suspected *Cryptosporidium* for six (3%), suspected *Legionella* for six (3%), suspected *Pseudomonas* for five (2%), suspected norovirus for two (1%), suspected *Giardia* for one (<1%), and unknown bacterial for one (<1%).

Hotels (i.e., hotels, motels, lodges, or inns) or resorts were associated with 71 (34%) of the 208 outbreaks; 50 (70%) of these outbreaks were associated with hot tubs. Among the 43 hotel- or resort-associated outbreaks with a confirmed etiology, 31 (72%) were caused by *Legionella* and were associated with a hot tub. Among the 208 outbreaks, 107 (51%) started during June–August (Figure 1). The June–August peak was driven by 63 outbreaks caused by *Cryptosporidium*; 58 (92%) of these outbreaks were associated with pools and seven (11%) with water playgrounds.^§^ One half (38) of the 76 outbreaks caused by *Cryptosporidium* occurred during 2016 (Figure 2). Twenty-six (13%) of the 208 outbreaks occurred during 2019.

**FIGURE 1:**
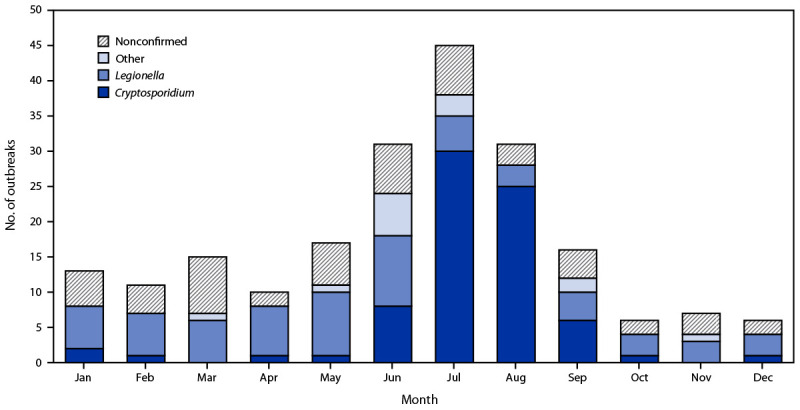
Outbreaks associated with treated recreational water* (N = 208), by etiology^†^^,^^§^ and month — National Outbreak Reporting System, United States, 2015–2019 * Treated recreational water is water in a pool, hot tub, water playground, or other artificially constructed structure that is intended for recreational or therapeutic purposes. Outbreaks are the occurrence of similar illness in two or more persons who are epidemiologically linked by location and time of exposure to 1) treated recreational water or 2) pathogens or chemicals that were aerosolized or volatilized into the air from treated recreational water. ^†^ “Nonconfirmed” includes outbreaks with the following reported etiologies: suspected chemical (e.g., excess chlorine, one or more disinfection byproducts, or altered pool chemistry), suspected *Cryptosporidium*, suspected *Giardia*, suspected *Legionella*, suspected norovirus, suspected *Pseudomonas*, unknown bacterial, and unknown. ^§^ “Other” includes outbreaks with the following confirmed etiologies: *Acanthamoeba, Campylobacter,* Shiga toxin–producing *Escherichia coli, Giardia*, nontuberculous mycobacteria, norovirus, or *Shigella*.

**FIGURE 2:**
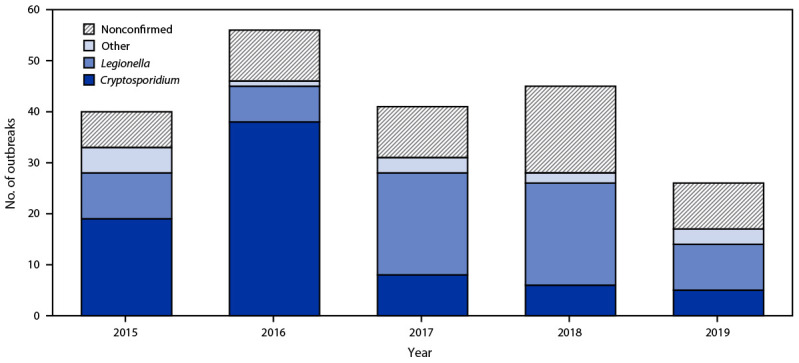
Outbreaks associated with treated recreational water* (N = 208), by etiology^†^^,^^§^ and year — National Outbreak Reporting System, United States, 2015–2019 * Treated recreational water is water in a pool, hot tub, water playground, or other artificially constructed structure that is intended for recreational or therapeutic purposes. Outbreaks are the occurrence of similar illness in two or more persons who are epidemiologically linked by location and time of exposure to 1) treated recreational water or 2) pathogens or chemicals that were aerosolized or volatilized into the air from treated recreational water. ^†^ “Nonconfirmed” includes outbreaks with the following reported etiologies: suspected chemical (e.g., excess chlorine, one or more disinfection byproducts, or altered pool chemistry), suspected Cryptosporidium, suspected Giardia, suspected Legionella, suspected norovirus, suspected Pseudomonas, unknown bacterial, and unknown. ^§^ “Other” includes outbreaks with the following confirmed etiologies: Acanthamoeba, Campylobacter, Shiga toxin–producing Escherichia coli, Giardia, nontuberculous mycobacteria, norovirus, or Shigella.

## Discussion

At least 208 outbreaks associated with treated recreational water occurred in the United States during 2015–2019. Most of these outbreaks were caused by *Cryptosporidium,* associated with pools, and started during June–August or were caused by *Legionella* and associated with hot tubs in hotels, motels, lodges, inns, or resorts. Outbreaks caused by *Cryptosporidium* can occur even if the pool or water playground is properly treated. Prevention steps beyond traditional operation, like those outlined in CDC’s 2018 MAHC (third edition) are needed to decrease the incidence of these outbreaks associated with public aquatic venues. Outbreaks caused by *Legionella* indicate that hot tub operation needs improvement, and taking steps as outlined in CDC’s MAHC, *Legionella* Control Toolkit,^¶^ and Water Management Program Toolkit** would decrease the incidence of these outbreaks associated with public hot tubs.

*Cryptosporidium* is transmitted when oocysts, the infectious life stage, are ingested (e.g., in contaminated recreational water). Oocysts are extremely tolerant to chlorine, the primary barrier to the transmission of pathogens in treated recreational water. At 1 ppm free available chlorine (*2*,*3*), oocysts can survive for >7 days in water, at pH 7.2–7.8^††^ and temperature 77°F (25°C). This is the minimum concentration recommended by CDC and typically required in U.S. jurisdictions for public aquatic venues. Because *Cryptosporidium* can persist in properly chlorinated water, it can cause larger outbreaks than those caused by pathogens that are inactivated within minutes by freely available chlorine at said concentrations and water pH and temperature. Other disinfection methods (e.g., ultraviolet light or ozone) have been found to be effective against oocysts (*4*,*5*). CDC’s 2018 MAHC recommends using these methods to achieve a minimum 3-log_10_ (99.9%) reduction of infectious oocysts in water playgrounds and a minimum 2-log_10_ (99%) reduction in all other aquatic venues (MAHC 4.7.3.3.2.1).^§§^The difference accounts for the substantially smaller volume of water in water playgrounds. In addition, water playgrounds are intended for young children aged <5 years, who have higher rates of cryptosporidiosis (*6*) and who sit on water playground jets and ingest recirculated, potentially fecally contaminated water from the jets (*7*).

When responding to diarrheal incidents (i.e., high-risk *Cryptosporidium* contamination events) in public pools or to cryptosporidiosis outbreaks associated with public pools, operators can follow the 2018 MAHC’s hyperchlorination^¶¶^ recommendations to inactivate oocysts. MAHC defines hyperchlorination as raising the free available chlorine to 20 ppm for 12.75 hours (MAHC 6.5.3.2) or, in the presence of ≤15 ppm cyanuric acid, 20 ppm free available chlorine for 28 hours (MAHC 6.5.3.2.1). Cyanuric acid is added to the water in outdoor pools to slow down the degradation of free available chlorine by the sun’s ultraviolet light; it does so by bonding with free available chlorine, consequently increasing the amount of time needed to inactivate *Cryptosporidium* (*3*) and other pathogens. The 2018 MAHC will be updated in 2021 with the release of the fourth edition. One proposed revision would establish parameters at which cyanuric acid concentration constitutes an imminent health hazard that requires immediate closure of a public aquatic venue pending correction. This would enable enforcement of maximum limits on the use of cyanuric acid.

*Legionella* is transmitted when aerosolized water droplets (e.g., droplets produced by hot tub jets) containing the bacteria are inhaled. *Legionella* can amplify when disinfectant concentration is not properly maintained, sediment or biofilm is present, water is not replaced frequently enough, or temperature is favorable (77–113°F [25–45°C]). Hot tubs operate in the temperature range that is favorable for *Legionella* growth (up to 104°F [40°C]), so maintaining disinfectant concentration, vigorously scrubbing all surfaces each time the hot tub is drained, and frequently replacing water are critical for *Legionella* control. These control measures are delineated in the *Legionella* Control Toolkit and the Water Management Program Toolkit Investigations of outbreaks caused by *Legionella* indicate that an effective water management program for hot tubs, as described in the toolkit, can reduce the risk of Legionnaires’ disease (*8*,*9*). Likewise, the 2018 MAHC recommends higher minimum disinfectant concentrations (3.0 ppm free available chlorine [MAHC 5.7.3.1.1.2.3] or 4.0 ppm bromine [MAHC 5.7.3.1.2.2]) than in other aquatic venues,*** not using cyanuric acid in hot tubs (MAHC 5.7.3.1.3.1), daily inspection for and removal of biofilm (MAHC 6.1.2.1.5.4), and regular water replacement (MAHC 5.12.1.2.1).^†††^ The 2018 MAHC also provides recommendations for disinfecting hot tubs associated with outbreaks caused by *Legionella*^§§§^ (MAHC 6.5.3.6.1).

The findings in this report are subject to at least four limitations. First, the outbreak counts presented are likely an underestimate of actual incidence. Many factors can present barriers to the detection, investigation, and reporting of outbreaks, such as voluntary reporting, lengthy incubation periods (e.g., of *Cryptosporidium*) and detection and investigation periods (e.g., of Legionnaires’ disease cases), and wide geographic dispersion of ill swimmers. Moreover, the public health response to the COVID-19 pandemic has been resource- and time-intensive. This circumstance could have been an additional barrier to 2020 efforts to finalize data on outbreaks that occurred during 2018 or 2019. Second, data might be skewed to include outbreaks of notifiable diseases (e.g., cryptosporidiosis and Legionnaires’ disease), cases of which are reported to and investigated by public health officials. Third, data on outbreaks with a chemical etiology might be limited because of the potentially transient nature of chemical contamination and potential lack of communication between those who respond to these outbreaks (e.g., hazardous materials personnel) and those who report them (e.g., infectious disease epidemiologists). Finally, data on factors contributing to the outbreaks were limited and could not be analyzed. Revisions to corresponding National Outbreak Reporting System data fields are underway to improve data quality and as part of data modernization efforts.

In addition to voluntarily adopting the MAHC and Legionnaires’ disease prevention recommendations, public health officials and operators of public aquatic venues can help prevent outbreaks associated with treated recreational water by educating the public. Given *Cryptosporidium*’s extreme chlorine tolerance, “don’t swim or let your kids swim if sick with diarrhea” and “don’t swallow the water you swim in” are important messages. The public can help prevent *Legionella* transmission by checking inspection scores, online or on-site, before getting in the water. The public can also conduct mini-inspections (e.g., measuring the bromine or chlorine level and pH with test strips available at most superstores, hardware stores, and pool supply stores) before getting into hot tubs. Persons at increased risk for Legionnaires’ disease^¶¶¶^ might choose to avoid hot tubs. These and other healthy swimming steps have been published.****

**Summary**

**What is already known about this topic?**

Outbreaks associated with treated recreational water in pools, hot tubs, and water playgrounds can be caused by pathogens or chemicals.

**What is added by this report?**

For the period 2015–2019, a total of 208 outbreaks associated with treated recreational water were reported to CDC. *Cryptosporidium* caused 76 outbreaks, resulting in 2,492 cases. *Legionella* caused 65 outbreaks, resulting in 13 deaths.

**What are the implications for public health practice?**

To help prevent outbreaks, operators of public aquatic venues and U.S. jurisdictions can voluntarily adopt CDC’s Model Aquatic Health Code, *Legionella* Control Toolkit, and Water Management Program Toolkit recommendations, and swimmers can follow CDC’s healthy swimming steps.

## Footnotes

* 45 C.F.R. part 46.102(l)(2); 21 C.F.R. part 56; 42 U.S.C. 241(d); 5 U.S.C. Sect. 552a; 44 U.S.C. 3501 et seq.

† Alabama, Alaska, Arizona, California, Colorado, Florida, Georgia, Hawaii, Idaho, Illinois, Iowa, Kansas, Kentucky, Louisiana, Maryland, Massachusetts, Michigan, Minnesota, Missouri, Nebraska, Nevada, New Hampshire, North Carolina, North Dakota, Ohio, Oklahoma, Oregon, Pennsylvania, South Carolina, Tennessee, Texas, Utah, Virginia, Washington, West Virginia, and Wisconsin.

§ Five cryptosporidiosis outbreaks were associated with both pools and water playgrounds.

¶
https://www.cdc.gov/legionella/wmp/control-toolkit/index.html

**
https://www.cdc.gov/legionella/wmp/toolkit

†† pH will determine the relative amounts of hypochlorous acid, the active disinfectant form of chlorine referred to as free available chlorine, and hypochlorite ion, a less active disinfectant form of chlorine. The pH range 7.2–7.8 is one that balances maximizing free available chlorine with swimmer comfort and preventing equipment corrosion.

§§ For reference purposes, MAHC elements discussed in this report are followed by the specific section number that corresponds to that element.

¶¶
https://www.cdc.gov/healthywater/swimming/aquatics-professionals/fecalresponse.html

*** The difference accounts for the depletion of the disinfectant concentration by higher water temperatures and aerosolization of water by hot tub jets.

††† The formula for calculating water replacement is frequency in days = (hot tub volume in gallons/3)/average number of users per day.

§§§
https://www.cdc.gov/legionella/downloads/hot-tub-disinfection.pdf

¶¶¶
https://www.cdc.gov/legionella/downloads/fs-legionnaires.pdf

****
https://www.cdc.gov/healthywater/swimming/swimmers/steps-healthy-swimming.html
